# Supplementary material for: Estimating pneumococcal carriage dynamics in adults living with HIV in a mature infant pneumococcal conjugate vaccine programme in Malawi, a modelling study
Source: BMC Med. 2024 Sep 27;22:419. doi: 10.1186/s12916-024-03631-5 (PMC11438070; doi:10.1186/s12916-024-03631-5)
Supplement: Supplementary file 1 — Additional file 1: Fig. S1 Susceptible-infected-susceptible (SIS) Markov model of pneumococcal carriage dynamics among Malawian adults living with and without human immunodeficiency virus (HIV) between 2021 and 2023. Table S1 Daily acquisition probability of vaccine-serotype (VT) and non-vaccine-serotype (NVT) pneumococcal carriage among adults living with and without HIV estimated from a Markov model. Table S2 Pneumococcal carriage duration (days) of vaccine-serotype (VT) and non-vaccine-serotype (NVT) among adults living with and without HIV estimated from a Markov model. Table S3 Daily pneumococcal serotype carriage acquisition probability and duration (days) of carriage of each serotype among ALWHIV and adult without HIV estimated from a Markov model. [file 12916_2024_3631_MOESM1_ESM.docx]

**Supplementary Information**

| 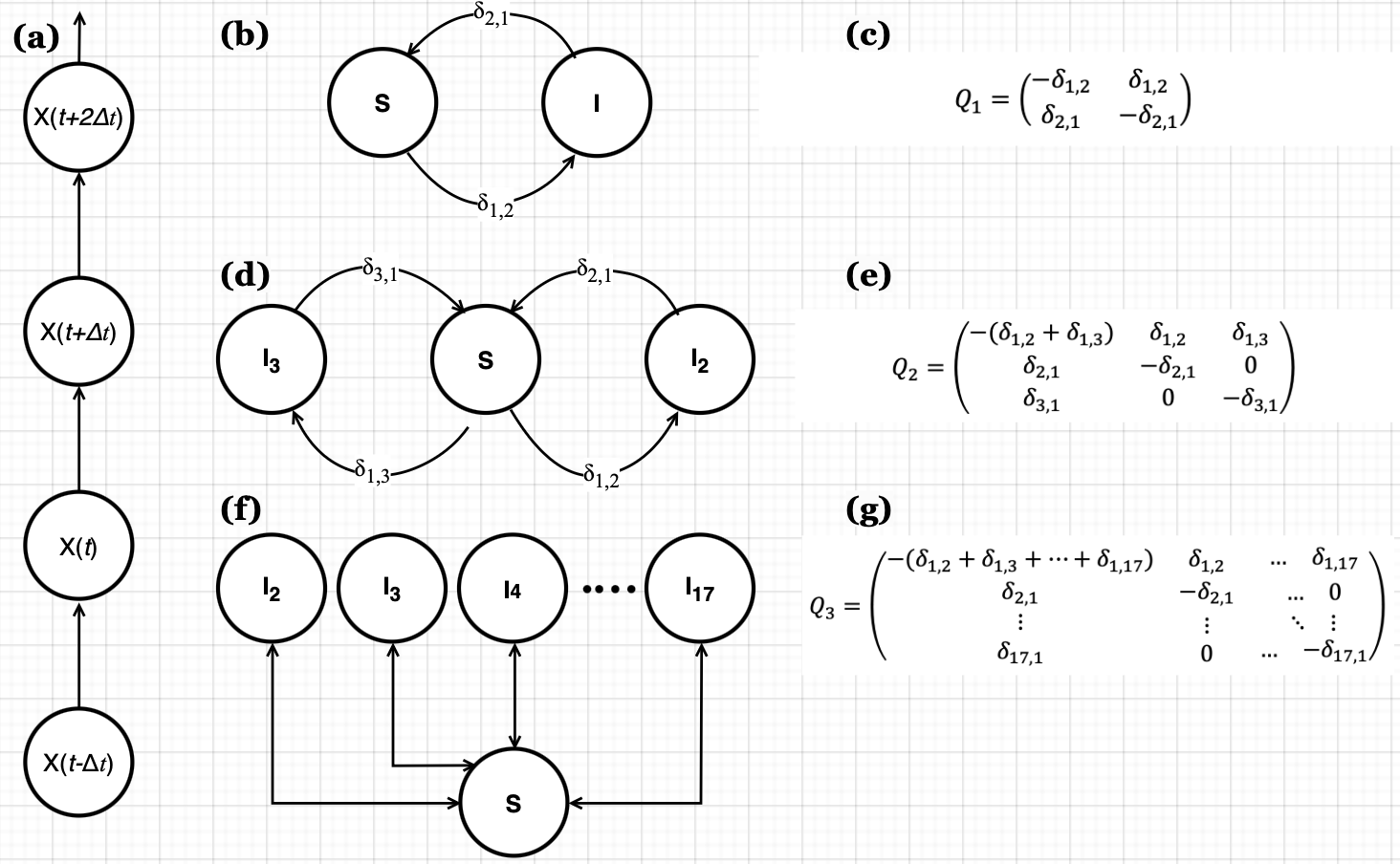 |
| --- |

**Supplementary Figure 1. Susceptible-infected-susceptible (SIS) Markov model of pneumococcal carriage dynamics among Malawian adults living with and without human immunodeficiency virus (HIV) between 2021 and 2023.** (a) Individual sequence of observed carriage states under a continuous-time changes ($\Delta t$) time-homogeneous intensities, where $X(t$) represents observed carriage state at time $t$ under a Markov property. (b) SIS model that captures the dynamics ($\delta_{ij}$) of whole pneumococcal nasopharynx carriage where ($I$) is carrier and ($S$) non-carrier states. (c) Transition intensity matrix ($Q_{1}$,) for carriage transitions between states to compute whole carriage rates ($\delta_{ij}$). (d) SIS model that captures the dynamics ($\delta_{ij}$) of pneumococcal nasopharynx carriage of pneumococcal conjugate vaccine (PCV13) serotypes ($I_{2}$), non-PCV13-serotypes ($I_{3}$) and non-carriers ($S$). (e) Transition intensity matrix ($Q_{2}$) for carriage transitions between states to compute vaccine serotype-group carriage rates ($\delta_{ij}$). (f) SIS model that captures serotype-specific dynamics ($\delta_{ij}$) of pneumococcal nasopharynx carriage of 16 serotypes including a few grouped PCV13 serotypes and non-PCV13 serotype ${(I}_{2}, I_{3}, I_{4}, \ldots I_{17})$. (g) Transition intensity matrix ($Q_{3}$) for carriage transitions between states to compute serotype-specific carriage rates ($\delta_{ij}$).

| **Supplementary Table 1.** **Daily acquisition probability of vaccine-serotype (VT) and non-vaccine-serotype (NVT) pneumococcal carriage among adults living with and without HIV estimated from a Markov model.** | | | | | | |
| --- | --- | --- | --- | --- | --- | --- |
|  | VT, HIV- | VT, ART<3m | VT, ART>1y | NVT, HIV- | NVT, ART<3m | NVT, ART>1y |
| Variable | Daily acquisition probability  (95%CI) | Daily acquisition probability  (95%CI) | Daily acquisition probability  (95%CI) | Daily acquisition probability  (95%CI) | Daily acquisition probability  (95%CI) | Daily acquisition probability  (95%CI) |
| Overall | 0.0022 (0.0011-0.0046) | 0.0014 (0.0006-0.0031) | 0.0022 (0.0010-0.0048) | 0.0142 (0.0101-0.0200) | 0.0192 (0.0135-0.0290) | 0.0205 (0.0143-0.0290) |
| Sex |  |  |  |  |  |  |
| Male | 0.0022 (0.0011-0.0046) | 0.0014 (0.0006-0.0031) | 0.0022 (0.0010-0.0048) | 0.0142 (0.0101-0.0200) | 0.0192 (0.0135-0.0290) | 0.0205 (0.0143-0.0290) |
| Female | 0.0042 (0.0021-0.0084) | 0.0027 (0.0012-0.0058) | 0.0044 (0.0020-0.0094) | 0.0216 (0.0158-0.0307) | 0.0294 (0.0202-0.0438) | 0.0312 (0.0222-0.0438) |
| Age group (years) |  |  |  |  |  |  |
| 18-33 | 0.0022 (0.0011-0.0046) | 0.0014 (0.0006-0.0031) | 0.0022 (0.0010-0.0048) | 0.0142 (0.0101-0.0200) | 0.0192 (0.0135-0.0290) | 0.0205 (0.0143-0.0290) |
| 34-44 | 0.0024 (0.0012-0.0047) | 0.0015 (0.0006-0.0033) | 0.0025 (0.0012-0.0049) | 0.0112 (0.0079-0.0158) | 0.0153 (0.0107-0.0220) | 0.0162 (0.0118-0.0222) |
| Number of children in the house |  |  |  |  |  |  |
| 1 child | 0.0022 (0.0011-0.0046) | 0.0014 (0.0006-0.0031) | 0.0022 (0.0010-0.0048) | 0.0142 (0.0101-0.0200) | 0.0192 (0.0135-0.0290) | 0.0205 (0.0143-0.0290) |
| 2+ children | 0.0038 (0.0017-0.0089) | 0.0024 (0.0010-0.0060) | 0.0040 (0.0017-0.0092) | 0.0156 (0.0105-0.0235) | 0.0212 (0.0143-0.0320) | 0.0225 (0.0154-0.0333) |
| SES |  |  |  |  |  |  |
| High | 0.0022 (0.0011-0.0046) | 0.0014 (0.0006-0.0031) | 0.0022 (0.0010-0.0048) | 0.0142 (0.0101-0.0200) | 0.0192 (0.0135-0.0290) | 0.0205 (0.0143-0.0290) |
| Low | 0.0044 (0.0020-0.0092) | 0.0028 (0.0014-0.0055) | 0.0046 (0.0024-0.0086) | 0.0194 (0.0138-0.0279) | 0.0264 (0.0193-0.0358) | 0.0280 (0.0206-0.0372) |
|  |  |  |  |  |  |  |
| 95%CI: Confidence intervals at significance level of 0.05  Cut off for age groups is based on the median age of 33 years-old  VT: serotypes targeted by 13-valency pneumococcal conjugate vaccine (PCV13)  NVT: serotypes not targeted by PCV13  ART: Antiretroviral therapy  HIV-: Adults living without human immunodeficiency virus  HIV+: Adults living with human immunodeficiency virus  SES: social economic status is based on possession index, calculated as a sum of positive responses for household ownership of each of one of fifteen different functioning items: watch, radio, bank account, iron (charcoal), sewing machine (electric), mobile phone, CD player, fan (electric), bednet, mattress, bed, bicycle, motorcycle, car, and television. | | | | | | |

| **Supplementary Table 2. Pneumococcal carriage duration (days) of vaccine-serotype (VT) and non-vaccine-serotype (NVT) among adults living with and without HIV estimated from a Markov model.** | | | | | | |
| --- | --- | --- | --- | --- | --- | --- |
|  | VT, HIV- | VT, ART<3m | VT, ART>1y | NVT, HIV- | NVT, ART<3m | NVT, ART>1y |
| Variable | Carriage duration  (95%CI) | Carriage duration  (95%CI) | Carriage duration  (95%CI) | Carriage duration  (95%CI) | Carriage duration  (95%CI) | Carriage duration  (95%CI) |
| Overall | 9.38 (5.49-15.96) | 17.85 (9.27-35.67) | 11.94 (6.44-22.02) | 9.99 (7.34-13.59) | 13.23(9.70-18.10) | 15.35 (11.71-20.41) |
| Sex |  |  |  |  |  |  |
| Male | 9.38 (5.49-15.96) | 17.85 (9.27-35.67) | 11.94 (6.44-22.02) | 9.99 (7.34-13.59) | 13.23 (9.70-18.10) | 15.35 (11.71-20.41) |
| Female | 10.04 (6.04-445.76) | 19.11 (9.58-37.31) | 12.79 (7.17-470.11) | 10.60 (8.04-14.02) | 14.04 (10.88-18.34) | 16.29 (12.49-21.55) |
| Age (years) |  |  |  |  |  |  |
| 18-33 | 9.38 (5.49-15.96) | 17.85 (9.27-35.67) | 11.94 (6.44-22.02) | 9.99 (7.34-13.59) | 13.23 (9.70-18.10) | 15.35 (11.71-20.41) |
| 34-44 | 7.20 (3.37-16.09) | 13.70 (7.22-25.37) | 9.17 (4.91-16.93) | 10.02 (7.22-13.77) | 13.27 (9.49-18.47) | 15.39 (11.69-20.61) |
| 95%CI: Confidence intervals at significance level of 0.05.  ART: Antiretroviral therapy.  NA: Not applicable for adults without human immunodeficiency deficiency virus.  HIV-: Human immunodeficiency deficiency virus negative infection status.  ALWHIVT: Human immunodeficiency deficiency virus positive infection status on antiretroviral therapy. | | | | | | |

| **Supplementary Table 3.** **Daily pneumococcal serotype carriage acquisition probability and duration (days) of carriage of each serotype among ALWHIV and adult without HIV estimated from a Markov model.** | | | |
| --- | --- | --- | --- |
| Serotype or serotype group | Proportion of total samples  n (%) | Acquisition probability  (95%CI) | Carriage duration  (95%CI) |
|  |  |  |  |
| 15A/B/C/F | 55 (5.7) | 0.0018 (0.0012-0.0027) | 15.82 (9.59-25.70) |
| 7A/B/C | 55 (5.7) | 0.0013 (0.0008-0.0021) | 17.83 (11.38-28.92) |
| 3 | 52 (5.4) | 0.0014 (0.0009-0.0023) | 13.32 (8.55-20.46) |
| 11A/B/C/D/F | 51 (5.3) | 0.0018 (0.0012-0.0028) | 12.18 (7.83-19.04) |
| 23A/B | 48 (5.0) | 0.0017 (0.0010-0.0026) | 9.02 (6.11-13.55) |
| 17A/F | 36 (3.7) | 0.0016 (0.0006-0.0018) | 15.30 (8.74-26.83) |
| 19F | 30 (3.1) | 0.0016 (0.0010-0.0026) | 8.57 (5.27-14.74) |
| 10A/B/C/F | 27 (2.8) | 0.0014 (0.0007-0.0023) | 7.80 (4.66-13.16) |
| 20 | 25 (2.6) | 0.0011 (0.0006-0.0019) | 9.71 (5.40-18.52) |
| 6C | 20 (2.1) | 0.0009 (0.0005-0.0016) | 10.74 (4.87-21.57) |
| 19A | 16 (1.7) | 0.0004 (0.0002-0.0009) | 17.38 (7.30-43.06) |
| 9A/L/N | 16 (1.7) | 0.0005 (0.0002-0.0013) | 9.23 (4.54-18.00) |
| 6A | 14 (1.5) | 0.0004 (0.0002-0.0010) | 12.94 (5.28-30.51) |
| Other VT | 45 (4.7) | 0.0016 (0.0010-0.0025) | 10.90 (6.96-17.34) |
| Known NVT | 64 (6.5) | 0.0023 (0.0016-0.0034) | 10.09 (8.61-11.74) |
| Unknown NVT | 410 (42.5) | 0.0165 (0.0141-0.0193) | 10.88 (7.39-16.40) |
| 95%CI: Confidence intervals at significance level of 0.05  Other VT: 13-valent Pneumococcal conjugate vaccine (PCV13) serotypes include 1, 4, 9V, 14, 18C, and 23F  known NVT: non-PCV13 serotypes include 22A/F, 33A/B/C/D/F, 18A/B/C/F, 12A/B/F, 19B/C, 8, and 6D | | | |
